# Pretreatment Source Location and Functional Connectivity Network Correlated With Therapy Response in Childhood Absence Epilepsy: A Magnetoencephalography Study

**DOI:** 10.3389/fneur.2021.692126

**Published:** 2021-08-03

**Authors:** Ke Zhang, Jintao Sun, Yulei Sun, Kai Niu, Pengfei Wang, Caiyun Wu, Qiqi Chen, Xiaoshan Wang

**Affiliations:** ^1^Department of Neurology, The Affiliated Brain Hospital of Nanjing Medical University, Nanjing Medical University, Nanjing, China; ^2^MEG Center, The Affiliated Brain Hospital of Nanjing Medical University, Nanjing, China

**Keywords:** childhood absence epilepsy, antiepileptic drug responders, antiepileptic drug nonresponders, ictal periods, source location, functional connectivity

## Abstract

**Objective:** This study aims to investigate the differences between antiepileptic drug (AED) responders and nonresponders among patients with childhood absence epilepsy (CAE) using magnetoencephalography (MEG) and to additionally evaluate whether the neuromagnetic signals of the brain neurons were correlated with the response to therapy.

**Methods:** Twenty-four drug-naïve patients were subjected to MEG under six frequency bandwidths during ictal periods. The source location and functional connectivity were analyzed using accumulated source imaging and correlation analysis, respectively. All patients were treated with appropriate AED, at least 1 year after their MEG recordings, their outcome was assessed, and they were consequently divided into responders and nonresponders.

**Results:** The source location of the nonresponders was mainly in the frontal cortex at a frequency range of 8–12 and 30–80 Hz, especially 8–12 Hz, while the source location of the nonresponders was mostly in the medial frontal cortex, which was chosen as the region of interest. The nonresponders showed strong positive local frontal connections and deficient anterior and posterior connections at 80–250 Hz.

**Conclusion:** The frontal cortex and especially the medial frontal cortex at α band might be relevant to AED-nonresponsive CAE patients. The local frontal positive epileptic network at 80–250 Hz in our study might further reveal underlying cerebral abnormalities even before treatment in CAE patients, which could cause them to be nonresponsive to AED. One single mechanism cannot explain AED resistance; the nonresponders may represent a subgroup of CAE who is refractory to several antiepileptic drugs.

## Introduction

Childhood absence epilepsy (CAE) is the most common idiopathic, generalized nonconvulsive epilepsy caused by multiple genetic etiologies, representing approximately 10% of pediatric epilepsy. This disease is characterized by brief moments of impaired consciousness and often occurs between the age of 3 and 8, affecting girls more than boys (1, 2). The typical ictal electroencephalography (EEG) shows 3- to 4-Hz generalized synchronous bilateral spike–wave discharges (GSWDs) (3).

CAE is recently considered as a network disorder, and the GSWDs are probably generated through the interconnection between the cortex and thalamic neurons (4). Furthermore, different changes in brain network among CAE patients occur, involving the default mode network (DMN), attention network, and salience network (5–7).

CAE patients are often treated with ethosuximide (ESM), lamotrigine (LTG), and valproic acid (VPA). Currently, ESM has class I evidence for CAE, and it is considered as the first line of treatment to cure it compared to LTG and VPA; however, ESM is unfortunately not available in China (8, 9). Although about two-thirds of patients recover completely, some continue to experience seizures or other psychosocial deficits into adulthood (10–12). Thus, early identification is necessary to plan a replacement therapy, patient counseling, individual support, and early referral. Despite many studies predicting the treatment outcome and prognostic factors in CAE patients (13–16), the reasons why some patients respond well to the treatment while others do not remain poorly understood. Potential causes of intractability include the induction of drug efflux transporters on the blood–brain barrier and the alteration of both the neurotransmitter receptors and target ion channels (17–19).

Magnetoencephalography (MEG) is a non-invasive method to detect the neuromagnetic signals of brain neurons, which is gradually applied in clinical practice (20, 21). MEG can localize epileptic activities and provide a dependable location in the cerebral cortex (22). Compared with functional magnetic resonance imaging (fMRI), MEG can measure at millisecond temporal resolution, which is useful for exploring epileptic neuromagnetic activities (23, 24). Different frequencies provide various temporal windows for processing, and various rhythms are correlated with distinct spatial scales (25). Sources in various frequency bands may uncover distinct corticothalamic networks in CAE patients (26).

Thus, based on the aforementioned background, in this work MEG was firstly used to study the ictal periods of absence epilepsy in untreated CAE patients, and then after the therapy, they were grouped according to their outcome, with the purpose of exploring the differences between antiepileptic drug (AED) responders and nonresponders. MEG was used to analyze the source location and functional connectivity network changes from low to high frequency in 24 drug-naïve CAE patients during ictal periods to investigate whether these alternations were correlated with the response to treatment. This study reveals new potential mechanisms involved in the treatment response of CAE patients, providing an indication for clinical treatment and prognosis.

## Materials and Methods

### Participants

A total of 46 CAE patients were monitored from 2014 to 2020. However, five patients were lost to follow up, 13 were treated with AEDs before the first MEG inspection, and four had no absence seizures during MEG recordings. Thus, 24 CAE patients were finally included in this study. All patients were accompanied by their parent or guardian who provided the informed consent on behalf of the child. The inclusion criteria were the following: (1) diagnosis of CAE by pediatricians according to the 2017 International League Against Seizure Classification (27), (2) an EEG showing 3 to 4 Hz GSWDs with at least 3 s of typical absence seizures, (3) head movement <5 mm during MEG recording, (4) normal neurological and physical condition, and (5) no history of AED treatment. The excluded criteria were the following: (1) presence of metal implants, (2) dramatic head movement and untypical absence seizures during MEG inspection, and (3) history of other neurological, psychiatric, or severe illnesses. During MEG recordings, eight patients were subjected to hyperventilation to evoke absence seizure, whereas the remaining patients had spontaneous absence seizures. A total of 24 patients with typical absence seizures were included in the subsequent analysis after removing inaccurate and incomplete seizures. All participants started the treatment with AEDs after the first MEG recordings as prescribed by the doctors of the Nanjing Children's Hospital in China. Clinical outcomes were evaluated at least 1 year after diagnosis by the parents' reports or EEG scans. The patients were then divided into two groups: one group consisting of AED-responsive patients and another group consisting of AED-nonresponsive patients. An AED-responsive patient was defined as one who had no clinically observed absence seizures and electroclinical discharges during video EEG recording along the follow-up. An AED-nonresponsive patient was defined as one who still had seizures after adequate doses of AEDs or who became seizure-free after the administration of more than two types of AEDs. Patients who became seizure-free after the administration of different types of AEDs due to adverse effects were considered as AED-responsive patients. The criteria were according to previously published papers (28, 29).

### MEG

The research data were acquired using the CTF-275-channel whole-head MEG system (VSM Medical Technology Company, Canada) at the magnetic shielding examination laboratory. Before collecting the MEG data, three coils were connected to the bilateral preauricular and nasion area, and the head location procedure was conducted before and after each collection to determine the patient's head locations, matching the MEG sensors prior to inspections. The audiovisual system was applied in monitoring the process. The sampling rate was 6,000 Hz, and the data were collected with noise elimination of third-order gradients. At least six epochs with a period of 2 min were collected for each patient, and then the patients were told to close their eyes and remain still during the scan. Head movement for each collection was restricted to 5 mm. If no ictal data were captured, another recording was performed after subjecting the patients to hyperventilate to trigger extra absence seizures.

### MRI

All patients were subjected to a T1-weighted image with a 3.0-T system magnetic resonance imaging (MRI) examination (Siemens, Germany). The parameters were as follows: the flip angle was 9°, the field of view was 250 × 250 mm, and the matrix was 512 × 512. We acquired 176 sagittal slices of each subject. The MRI marks were placed in the same position as those used in the MEG inspection for a precise co-registration of the MEG and MRI datasets. All the anatomical parts in the MRI could be recognized in the digital MEG images.

### MEG Data Analysis and Signal Processing Technology

**Figure 1:**
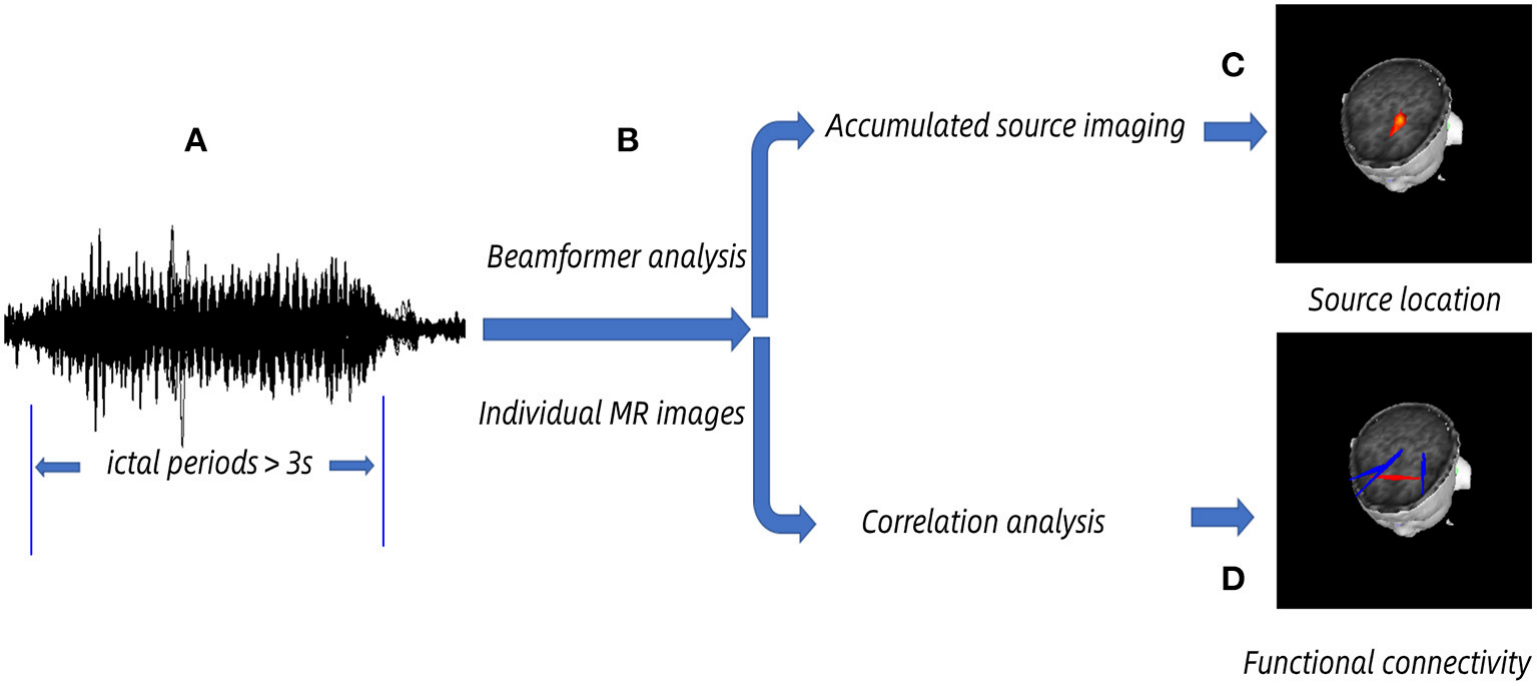
Schematic diagram of magnetoencephalography (MEG) data analysis. MEG data were acquired, and we chose >3 s ictal periods for analysis. **(A)** Ictal periods. **(B)** The beam-former was applied to cast sensor-level signals to the anatomical brain regions according to individual MR images at the source level. **(C)** Accumulated source imaging was generated by spatially overlying the source MEG data and exhibiting the predominant source location. **(D)** Functional connectivity network was obtained by correlation analysis, and the significant network was acquired at a predefined threshold.

MEG data were filtered by a 1–4-Hz band pass filter after removing noticeable magnetic artifacts and noise (amplitude >6 pT). The selection of ictal periods was based on 3-Hz GSWDs shown in MEG and clinically observed symptoms. The clinical manifestations were observed by the audiovisual system. The epileptic waveforms were recognized by a specialist neurological physician. The first spike wave and the last slow wave in GSWDs were defined as the beginning and the ending of the seizure separately. An ictal segment of more than 3 s for data processing was chosen (Figure 1). The MEG signals of predefined six frequency bands at 1–4, 4–8, 8–12, 12–30, 30–80, and 80–250 Hz were analyzed, and the power-line noise at 50 Hz was avoided.

### Source Localization

According to the previously published paper (30), accumulated source imaging (ASI) technique was used to sum the volumes of source activities over a period of time with two-step beamforming methods, which could quantitatively compare the neuromagnetic activity between responders and nonresponders. The ASI was applied in separating correlated sources with multiple source beamformers. The sources were located by ASI using node-beam lead fields. Because each node-beam lead field represented a form of either subspace solution or source-beamformer (30), the entire brain was scanned at a resolution 6 mm (about 17,160 voxels per magnetic source). ASI was obtained using the following formula:

$$\begin{array}{l} Asi\left(r,s\right)=\overset{t=n}{\underset{t=1}{\sum }}Q\left(r,t\right) \end{array}$$

where Asi stands for the accumulative source strength at location *r, s* represents time slice, *t* represents the time point of MEG data, *n* stands for the entire time points of MEG data, and *Q* is the source activity at the time point *t* and source *r*. Detailed algorithms were described in the previously published paper (30), and this method has been used in several research (31–36).

### Functional Connectivity

According to previous studies (30, 35, 36), the ASI algorithms and the correlation analysis were used to elaborate the functional connectivity network between the responders and nonresponders at the source level. The volumetric sources of activities in the whole brain were firstly calculated using individual MR images (31). The signal correlation of virtual sensors from two source pairs was analyzed to estimate the neural networks during the selected ictal period. The correlativity between the virtual sensor signals from the two source pairs was analyzed by calculating the correlation coefficient, and the correlation coefficient was expressed as per the following formula:

$$\begin{array}{l} R\left(xa,xb\right)=\frac{C\left(xa,xb\right)}{SxaSxb} \end{array}$$

where *R*(*xa, xb*) represents the correlation between two magnetic sources at position “*a*” and “*b*,” *xa*, and *xb* are the signals from the paired magnetic sources to calculate the correlation, *C*(*xa, xb*) represents the average value of the signals from the paired magnetic sources, and *Sxa* and *Sxb* are the standard deviations of two source signals.

All possible links between every two source pairs were evaluated at the source level to avoid bias, and the neural network distribution was superimposed on the individual MR images (30, 31). Individual MR images were spatially normalized using an MRI template (37, 38), and the neural networks were overlaid on individual MR images. Then, the MEG–MRI data were spatially normalized to the MRI template. The medial frontal cortex (MFC) was specially selected as the region of interest (ROI). The ROI was firstly defined visually and then verified with coordinates through the MRI template. The neural networks between responders and nonresponders were visually recognized in MRI views and displayed in the coronal, axial, and sagittal positions. The midpoint of the right and left preauricular points was the original point (*x* = 0, *y* = 0, and *z* = 0). The blue and red colors indicated the negative and positive connections, respectively. Our MEG processor software could segment and visualize the brain regions in 2D and 3D views.

The threshold was defined as a checkpoint to guarantee the data quality. The *t* values for the entire magnetic source pairs were used to identify the threshold for the connection and calculated using the following equation:

$$\begin{array}{l} Tp=R\sqrt{\frac{K-2}{1-R^{2}}} \end{array}$$

where *Tp* represents the *t* value of a correlation, *R* stands for the correlation of magnetic source pairs, and *K* represents the number of connected data points. This study specifies the *Tp* value when *p* < 0.05 was defined as the threshold of functional connectivity network within the two groups.

The algorithms discussed above were analyzed through MEG Processor software (http://sites.google.com/site/braincloudx/). The detailed mathematical algorithms were described in previous studies (30, 31), and this method has been used in several research (35, 36, 39).

### Statistical Analyses

The magnetic source location and functional connectivity under six frequency bandwidths were compared between AED-responsive patients and AED-nonresponsive patients using Fisher's test. The clinical data of the patients (age of onset, average age, follow-up time, and duration of absence seizures during MEG recording) between responders and nonresponders were analyzed using two-tailed *t*-test, and the results were represented as mean ± SD. The types of AED between the two groups were analyzed using Fisher's test. A value of *p* < 0.05 was considered statistically significant. Multiple comparisons were corrected by Bonferroni-corrected methods [i.e., for six frequency bands, *p* < 0.0083 (0.05/6 = 0.0083)]. Controlling the false discovery rate could be utilized to solve the problem of type 1 errors (40). Statistical analysis was performed by SPSS 25.0 (IBM SPSS Inc., USA).

## Results

### Subject Characteristics, Therapy, and Therapeutic Response

The gender ratio among patients was 4:20 (male/female). Thirteen patients were responsive, and 11 were nonresponsive. Among the responders, LTG was administered to nine patients, and VPA was administered to the other four. All the responders became seizure-free. Among the nonresponders, LTG was administered to two patients, VPA was administered to five, and a combination of VPA–LTG was administered to the other four. The seven nonresponders treated with monotherapy still had seizures, whereas the four treated with combination therapy became seizure-free. The average age of the 24 patients was 10.28 ± 2.82 years; the onset age was 6.29 ± 1.33 years, with a mean duration of the seizure of 14.10 ± 4.70 s and a follow-up time of 28.67 ± 22.07 months. The clinical characteristics of the responders and nonresponders were not significantly different (onset age, average age, age at the time of diagnosis, gender, seizure durations, follow-up time, and types of AEDs). The clinical data are shown in Table 1.

**Table 1:** Characteristics of the patients.

**Patient**	**Gender/age (years)**	**Onset age (years)**	**Seizure durations (s)**	**Follow-up time (months)**	**Initial aed**	**Aeds added**	**Seizure-free**
1^a^	F/8	6	16.5	12	VPA	N	Y
2^a^	F/9	6	10	23	LTG	N	Y
3^a^	F/8	5	7.1	20	LTG	N	Y
4^a^	F/9	6	7.2	20	LTG	N	Y
5^a^	F/10	8	14.4	20	LTG	N	Y
6^a^	F/13	7	15	68	LTG	N	Y
7^a^	F/11	5	15.5	74	VPA	N	Y
8^a^	F/8	6	5	23	LTG	N	Y
9^a^	F/13	7	14.8	68	LTG	N	Y
10^a^	F/8	7	24	13	LTG	N	Y
11^a^	M/6	5	10.2	14	VPA	N	Y
12^a^	F/7	6	14.7	13	LTG	N	Y
13^a^	M/8	7	18	12	VPA	N	Y
14^b^	M/14	6	20	24	VPA	N	N
15^b^	F/15	8	18.5	25	LTG	VPA	Y
16^b^	F/9	7	9.3	12	LTG	N	N
17^b^	F/10	8	15	26	VPA	N	N
18^b^	F/10	5	11.2	15	VPA	N	N
19^b^	F/11	10	11.3	12	LTG	N	N
20^b^	F/14	4	11.3	67	LTG	VPA	Y
21^b^	M/11	6	18	23	LTG	VPA	Y
22^b^	F/11	5	13	13	VPA	N	N
23^b^	M/7	5	20.5	19	VPA	N	N
24^b^	F/17	6	18	72	LTG	VPA	Y

*F, female; M, male; Y, yes; N, no; aeds, antiepileptic drugs; LTG, lamotrigine; VPA, valproic acid*.

a *mean responder*.

b *mean nonresponder*.

### Source Location

**Figure 2:**
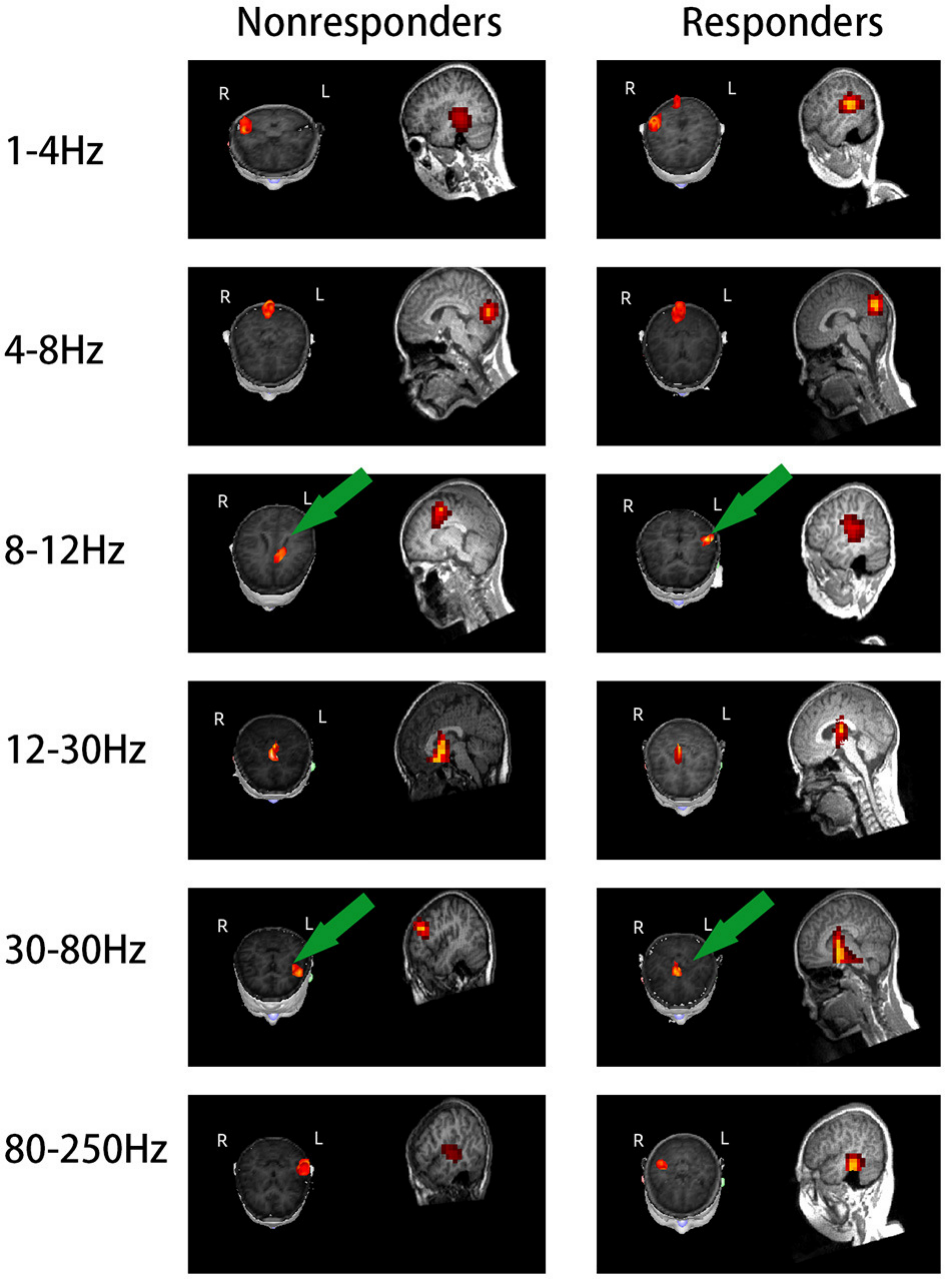
Images of magnetic localization at 1–250 Hz frequency band. Significant differences were labeled with a green arrow. The location in the nonresponders was mainly in the frontal cortex area at 8–12 and 30–80 Hz, especially at 8–12 Hz, as opposite to the responders; the nonresponders showed activation in the medial frontal cortex area.

**Figure 3:**
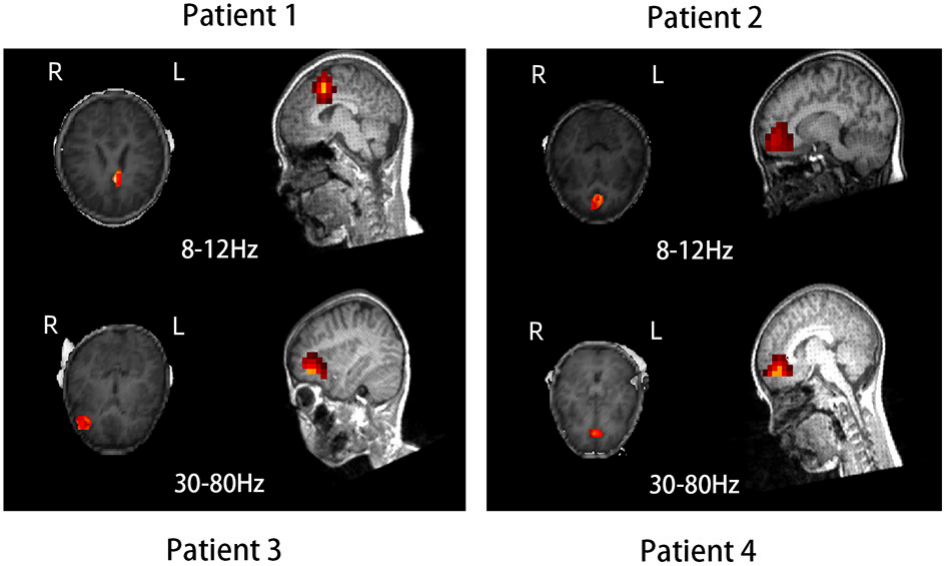
Source location in the dorsal medial frontal cortex (patient 1), medial prefrontal cortex (patients 2 and 4), and lateral frontal cortex (patient 3) in four typical nonresponders at 8–12 and 30–80 Hz.

The source location of the nonresponders was mainly in the frontal cortex at 8–12 and 30–80 Hz, especially 8–12 Hz. The source location of the nonresponders was mostly in the medial frontal cortex, which was chosen as the region of interest (*p* = 0.005, *p* < 0.0083; Figure 2 and Table 2). In addition, it is important to note that majority of the areas of MFC were the medial prefrontal cortex (MPFC) and dorsal medial frontal cortex (DMFC) (Figure 3), while in the other four frequency bands, the two groups were not statistically different.

**Table 2:** Magnetic location in the brain of the responders and nonresponders.

**Frequency band (Hz)**	**1–4**		**4–8**		**8–12**		**12–30**		**30–80**		**80–250**	
**Group**	**N**	**R**	**N**	**R**	**N**	**R**	**N**	**R**	**N**	**R**	**N**	**R**
FC	4	3	4	1	7^*^	0^*^	3	2	10^*^	1^*^	3	1
(MFC)	3	3	4	1	7^*^	0^*^	3	2	5	0	3	0
(LFL)	1	0	0	0	0	0	0	0	5	1	0	1
TC	1	0	1	0	1	2	0	2	0	3	3	6
TPJ	1	2	0	0	0	3	0	1	0	0	0	1
POT	6	5	4	2	2	2	3	2	0	0	0	0
Pc	0	3	1	0	0	1	2	3	1	1	0	0
PCC	0	2	0	0	0	0	2	0	0	1	0	0
PL	1	1	2	1	2	1	1	0	0	0	0	0
MOC	2	4	0	2	1	2	1	2	0	1	0	0
TH	0	3	1	4	1	1	3	3	2	7	1	1
CE	0	0	1	0	0	0	0	0	0	0	1	2
DBA	0	1	0	2	0	2	1	0	3	1	4	3

*N, nonresponders; R, responders; FC, frontal cortex; LFL, lateral frontal lobe; MFC, medial frontal cortex; TPJ, temporal–parietal junction; POT, parietal–occipital–temporal junction; pC, precuneus; PCC, posterior cingulate cortex; PL, parietal lobe; MOC, medial occipital cortex; TH, thalamus; CE, cerebellum; DBA, deep brain area*.

* *p < 0.05*.

### Functional Connectivity

**Figure 4:**
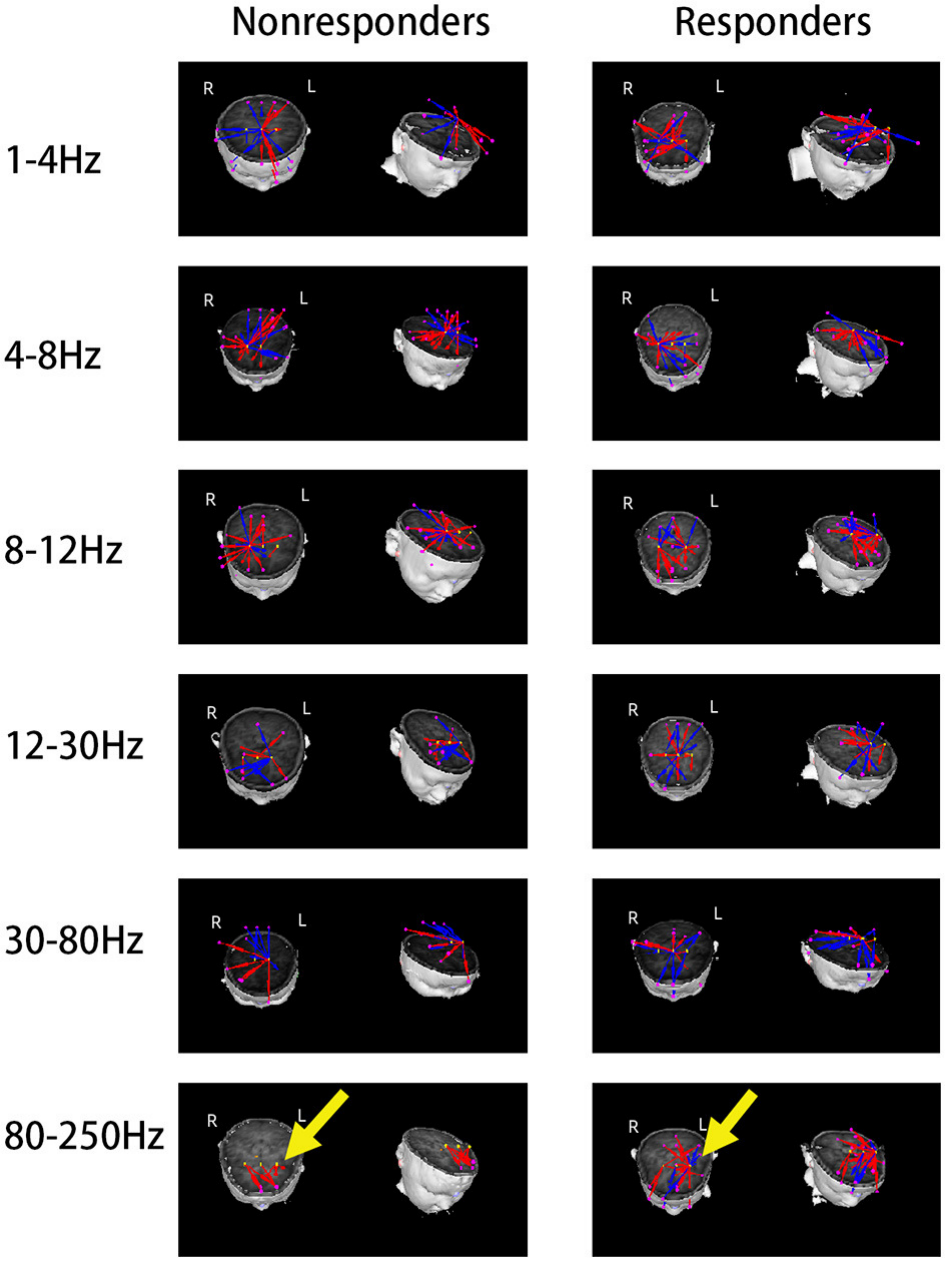
Typical predominant functional connectivity networks at 1–250 Hz. Nonresponders showed a significantly altered functional connectivity network at 80–250 Hz as compared to the responders, represented by local frontal connections and deficient anterior–posterior connections, which were indicated by yellow arrows.

Because the source location of the nonresponders was mainly located in the MFC at 8–12 Hz, our hypothesis was that the network involving MFC could be different between the two groups. Thus, MFC was chosen as the ROI. The results were co-registered to each patient's brain MR images using the three fiducial points, and visual inspection was conducted in both 2D and 3D views by two specialist neurological physicians independently. Both excitatory and inhibitory connections were analyzed. The functional connectivity was significantly different between the responders and nonresponders (*p* < 0.0001; Figure 4) at 80–250 Hz. At 80–250 Hz, the nonresponders showed strong positive connections in the frontal cortex (excluding other brain areas) compared with the responders. Nevertheless, both the responders and nonresponders showed excitatory connections between the anterior and posterior brain areas in the other five frequency bandwidths, and no significant differences were observed between the two groups in these frequency bands.

## Discussion

Our study using MEG revealed that the differences in pretreatment source location and functional connectivity network between AED responders and AED nonresponders were frequency dependent, supporting the existence of new mechanisms of AED response in CAE children. However, the clinical characteristics between the responders and nonresponders did not change significantly in our study.

### Source Localization

Among the nonresponders, an evident frontal cortex source location at 8–12 and 30–80 Hz, especially the MFC (including MPFC and DMFC) source location at 8–12 Hz was found compared with the responders.

The frontal cortex has numerous high-order functions (41–43), and some studies reported that the orbital/lateral frontal lobe may be of importance for CAE pathophysiology (44, 45). The lower frontal myelin water content in CAE patients reflects an altered neurodevelopment, and the frontal lobe regions are most closely related to the cortical thickness of the entire brain in CAE patients (46, 47). Moreover, to the best of our knowledge and contrary to popular belief, cortical oscillations, instead of sole thalamic rhythmicity, are pivotal in inducing epilepsy and establishing a spike–wave discharge frequency (48), and the frontal cortex is of great importance in initiating and spreading absence epilepsy (44, 49–51).

However, the subgroups of CAE patients related to AED response are insufficiently studied. A previous EEG study revealed that the frontal onset of absence seizures is sometimes harder to control with traditional AED monotherapy and can be considered a special subtype (52). P. W. Carney used EEG–fMRI to classify the diverse individual patterns of frontal cortical activation associated with absence seizures, and they postulated that the involvement of dorsolateral prefrontal cortex in this disease is highly associated to cognitive impairment in CAE, and the group with a primarily negative signal change in the dorsolateral prefrontal cortex has a more benign phenotype compared to the positive group (53).

Furthermore, in our study, the MFC mainly included MPFC and DMFC. MPFC is an important hub of default mode network, which is closely connected to the limbic system (54) and is considered abnormal in CAE patients (5, 55). Thus, it is likely that source locations involving these crucial hubs potentially reveal dysfunctional intrinsic activities and can make CAE patients become more intractable.

The frequency band where the source was located was mainly at 8–12 and 30–80 Hz, both expressing low-frequency brain activities. The α rhythm (8–12 Hz) is an obvious oscillation during wakefulness and relaxation (56). A study has shown that idiopathic, generalized epilepsy patients represent increased functional connectivity in low-α frequencies (57). The γ rhythm (30–80 Hz) is important in information processing within cortical networks (58). A research found that the shift in γ power (30–80 Hz) was associated with AED efficacy in two absence epilepsy mouse models (59). Notably, we observed nonresponders located in MFC at 8–12 Hz compared with the responders in our study. A previous study using EEG found that α-power shifted in intractable epilepsy patients, which might reflect dysfunction of a large-scale cortico-thalamic network, including frontal cortex (60). Various frequency bands prefer diverse sorts of connections and different spatial and temporal information integrations. Low-frequency oscillators include numerous groups of neurons in large cerebrum regions as compared with high-frequency oscillators (HFOs) (61). Thus, the source of low-frequency brain activity may not always result in a description of potential epileptogenic zones (33).

Overall, our results made preliminary assumptions that the frontal lobe and especially the medial frontal lobe at α band might be relevant to AED-nonresponsive CAE patients.

### Network Pattern

Notably, the source location does not represent the aberrant brain tissue but indicates the area involved in a dynamic network of discharges (62). Epileptic seizures reflect aberrant synchronization, which can only be learnt at the level of neuronal networks (63). Functional connectivity is considered the statistical dependencies among distant neurophysiology events (64). The network perspective assists in understanding the epileptogenesis of epilepsy, which may result in the progression and maintenance of intractable or chronic epilepsy (65).

Therefore, given the importance of MFC that we discovered initially and the advantages of functional connectivity networks, the dynamics of functional connectivity networks involving the MFC were specially studied.

Our results identified a stronger positive functional connectivity in local frontal cortex at 80–250 Hz in nonresponders. Fair DA supposed that, as the brain grows, the connections between the anterior and posterior cortices are strengthened (66). However, the increased connection within the frontal cortex and the decreased connection between the anterior and posterior cortex might not simply be due to immature brain development. A previous study has demonstrated increased functional connectivity within the orbitofrontal cortex in CAE patients (67). Moreover, the network-involved MFC presented decreased connections with other regions. The MFC was a hub of DMN, which was revealed to be abnormal in CAE patients (5). A previous study revealed reduced DMN connectivity in treatment-resistant idiopathic, generalized epilepsy (68). Intractable epilepsy may arise from the start rather than evolve over time, and clinical symptoms are obvious in the early stage of the disease (69). Thus, we speculated that this local frontal connection found in our study might be caused by underlying cerebral abnormalities even before treatment in CAE patients, potentially leading to them to become AED nonresponders, but in our study, given the small sample size of the two groups, we could not definitely extend our results to the entire CAE patient population. Further study is needed in the future.

In our study, the CAE patients were treated with VPA or LTG rather than ESM because ESM is not available in China. The mechanisms used by VPA and LTG to resolve CAE were unclear. Previous studies concluded that T-type calcium channels in nucleus reticularis and cortical neurons are vital in absence seizure expression, which is consistent with the gain-of-function mutation of the Cav3.2 subtype of T-type calcium channels appearing in some CAE patients (70, 71). This evidence might explain the weak and non-selective action of VPA on these channels, which leads to its ineffectiveness (72). In addition, LTG is generally considered as a member of the sodium channel blockers of AEDs, but this mechanism is not explaining its current clinical effect on controlling absence seizures (73, 74). Overexpression of drug efflux transporters in epilepsy, mostly P-glycoprotein, at the blood–brain barrier can be involved in LTG resistance (75, 76). However, the relationship between the pretreatment neural network during ictal periods found in our study and the molecular mechanisms discussed above is unknown. Thus, further studies are needed to investigate the underlying connections between them.

A study speculated that the receptors used by VPA to exert its effect are spread in all parts of the corticoreticular network, potentially explaining the effect of this AED (77). A previous study evaluating the therapy outcome in CAE demonstrated that the involvement of post-dorsal lateral medial frontal cortex in CAE patients may be the reason of the initial LTG therapy failure, and VPA effectiveness can be associated with the complexity of the neural network (78). Furthermore, a study using a combined EEG–fMRI and MEG analysis revealed that CAE nonresponders have a pretreatment increased connectivity in the frontal cortex, potentially leading to the absence of the effect of ESM on the thalamus in comparison with the effect of ESM treatment in responders (29).

Whereas, in our study changes in the local medial frontal cortex network were discovered in nonresponders, independently on the type of AED the children received, our results potentially suggest that the pretreatment network differences between responders and nonresponders were not specific to one single AED but represented a subgroup of CAE who was refractory to any medical treatment, an aspect that is in agreement with the previous study discussed above (29).

The significant changes of functional connectivity networks were at 80–250 Hz. HFOs are usually divided into ripples (80–250 Hz) and fast ripples (250–500 Hz), which are appropriate for detecting adjacent and strongly connected neural connections and may offer accurate spatial information about cortical malfunction (61). It has been uncovered that ictal HFOs are produced by the disorganization of neural firing, which are highly located in epileptogenic zones (79). Tenney indicated that ictal HFOs prominently appeared in the frontal area in CAE patients (26), and a previous study of our group reported that the ictal HFOs reflected the severity of absence seizures (33). HFOs might also serve for monitoring AED response (80).

To sum up, our results display the abnormal dynamic networks involving the MFC in nonresponders at 80–250 Hz, which might further reveal underlying cerebral abnormalities even before treatment in CAE patients, leading to nonresponsive AEDs.

Nevertheless, the response ratio of the treatment in CAE patients varied from 60 to 95%, which was depending on different factors, including the studied population, the measurement of the outcomes, the length of the follow-up, pretreatment EEG semiology, genetic abnormalities, and different AED mechanisms (15, 16, 81–83). It is probably an understatement to assume that AED resistance was caused by a single mechanism because it could be due to multiple factors coexisting in the same patient.

## Limitations

Our research has certain limitations. Firstly, the clinical data were provided by the parents, potentially resulting in inexact information. Secondly, given the strict inclusion criteria, our analysis is limited to a relatively small sample size; thus, it is not possible to draw any definitive conclusions, and further studies are needed to confirm our results. Thirdly, the relationship between cortical multi-frequency pretreatment neuromagnetic activities and the underlying molecular mechanisms remained unknown. Finally, the repeatability and reliability of our measurements remain to be proven. Therefore, further research is needed to confirm the underlying mechanisms of AED response in CAE patients.

## Conclusions

Our study demonstrated the pretreatment source location and functional connectivity network associated with the different treatment responses in CAE patients. The frontal cortex and especially the MFC at α band might be relevant to AED-nonresponsive CAE patients. The local frontal positive connection at a high-frequency range in our study might be probably caused by underlying cerebral abnormalities even before treatment in CAE patients, leading to nonresponsive AEDs. It is probably an understatement to assume that one single mechanism was responsible for AED resistance; the nonresponders might represent a subgroup of CAE who were refractory to several antiepileptic drugs. Thus, the specific mechanisms underlying the treatment response need to be further investigated.

## Data Availability Statement

The original contributions presented in the study are included in the article/supplementary material, further inquiries can be directed to the corresponding author/s.

## Ethics Statement

The studies involving human participants were reviewed and approved by The Affiliated Brain Hospital of Nanjing Medical University. Written informed consent to participate in this study was provided by the participants' legal guardian/next of kin. Written informed consent was obtained from the individual(s), and minor(s)' legal guardian/next of kin, for the publication of any potentially identifiable images or data included in this article.

## Author Contributions

KZ analyzed the data and wrote this manuscript. JS and YS contributed to data analysis. KN, PW, and CW recruited patients. QC recorded the MEG data. All authors agreed to publish this manuscript.

## Conflict of Interest

The authors declare that the research was conducted in the absence of any commercial or financial relationships that could be construed as a potential conflict of interest.

## Publisher's Note

All claims expressed in this article are solely those of the authors and do not necessarily represent those of their affiliated organizations, or those of the publisher, the editors and the reviewers. Any product that may be evaluated in this article, or claim that may be made by its manufacturer, is not guaranteed or endorsed by the publisher.
